# Ultramarathon is an outstanding model for the study of adaptive responses to extreme load and stress

**DOI:** 10.1186/1741-7015-10-77

**Published:** 2012-07-19

**Authors:** Grégoire P Millet, Guillaume Y Millet

**Affiliations:** 1 ISSUL Institute of Sport Sciences, Department of Physiology, Faculty of Biology and Medicine, University of Lausanne, Switzerland; 2 Université de Lyon, F-42023, Saint Etienne, France

**Keywords:** Cerebral adaptations, extreme environment, overload pathologies, ultra-endurance

## Abstract

Ultramarathons comprise any sporting event involving running longer than the traditional marathon length of 42.195 km (26.2 miles). Studies on ultramarathon participants can investigate the acute consequences of ultra-endurance exercise on inflammation and cardiovascular or renal consequences, as well as endocrine/energetic aspects, and examine the tissue recovery process over several days of extreme physical load. In a study published in *BMC Medicine*, Schütz *et al. *followed 44 ultramarathon runners over 4,487 km from South Italy to North Cape, Norway (the Trans Europe Foot Race 2009) and recorded daily sets of data from magnetic resonance imaging, psychometric, body composition and biological measurements. The findings will allow us to better understand the timecourse of degeneration/regeneration of some lower leg tissues such as knee joint cartilage, to differentiate running-induced from age-induced pathologies (for example, retropatelar arthritis) and finally to assess the interindividual susceptibility to injuries. Moreover, it will also provide new information about the complex interplay between cerebral adaptations/alterations and hormonal influences resulting from endurance exercise and provide data on the dose-response relationship between exercise and brain structure/function. Overall, this study represents a unique attempt to investigate the limits of the adaptive response of human bodies.

Please see related article: http://www.biomedcentral.com/1741-7015/10/78

## Background

While the industrialized world adopts a sedentary lifestyle, ultramarathon running races have become increasingly popular in the last few years, notably in the US, Europe, Japan, Korea, and South Africa. The ability to run long distances is also considered to have played a role in human evolution [1]. This makes the issue of ultra-long distance physiology relevant. Ultramarathons are basically either performed on mostly flat roads or tracks, or run on varied terrain trails. They comprise races that are completed over the space of multiple days (for example, 6 days), with the winner being the one that covers the most distance within this set period of time or races that cover a specified distance during a single stage, which normally range from 50 km to 160.9 km or over several stages. The paper by Schütz *et al. *[2] explores the physiological changes that occur in runners during this latter type of events, probably one of the most demanding physical exercise in humans, maybe only overpassed by polar expeditions (for example, Scott's party man-hauling their sleds across the Antarctic for 159 days in 1911/1912, [3]) or other individual challenges such as the run around Europe in 2009/2010, that is, 27,012 km in 1 year (74 km/day), by the Frenchman Serge Girard. We recently reviewed the origin of muscle fatigue after prolonged exercises lasting from 30 minutes to several hours [4] and found that the knee extensors isometric strength loss increased in a non-linear way with exercise duration, that is, there was a plateau after approximately 20 h of running. Recent findings from our group confirm this result for an even longer mountain ultramarathon (Tor des Geants, 330 km; unpublished results). These fatigue studies and other experiments conducted on inflammation [5], cardiovascular or renal consequences [6,7], endocrine/energetic aspects (see for example [8]) help to understand acute consequences of an ultra-endurance exercise (generally shorter than 24 h, more rarely 2 or 6 days) but do allow examining recovery process, that is, tissue degeneration/regeneration over several days of extreme physical load.

## Trans Europe Foot Race: studying the limits of human endurance

In an observational cohort study on 44 ultramarathon runners over 4,487 km in 64 stages from South Italy to North Cape, Norway (the Trans Europe Foot Race 2009), Schütz *et al. *[2] recorded daily sets of data from magnetic resonance imaging (MRI), psychometric, body composition and biological measurements. Beyond the logistical achievement of following the runners and moving a 30-m, 45-tonne 1.5 Tesla whole-body MRI across Europe (!), they succeeded with a high rate of test completion and data collection.

This 'field' experiment is unique since it is impossible to expect subjects pushing to (and sometimes beyond) their limits for 64 days without any day of rest in a laboratory setting. Such commitment can be achieved only in an official competition and is absolutely necessary for exploring the adaptive responses in healthy subjects at the limit of stress.

In our view, in the field of sports medicine, the longitudinal design of this study will allow us to better understand the time course of degeneration/regeneration of some lower leg tissues as knee joint cartilage or ventral tibial periosteum, to describe the adaptive responses (for example, red bone marrow hyperplasia), to differentiate running-induced from age-induced pathologies (for example, retropatelar arthritis), to understand why some painful reactions (for example, 'shin splints') can be 'over-run' whereas others lead to severe injuries (for example, stress fracture) and finally to assess the interindividual susceptibility to injuries.

This study will also bring new information about the complex interplay between cerebral adaptations and hormonal influences resulting from endurance exercise. To date, it is known that moderate exercise is beneficial to brain heath (for example, increased perfusion or increased brain-derived neurotrophic factor (BDNF)) [9,10]. But the potential deleterious effects (for example, atrophy, ischemia, brain lesions) of extreme loads on brain volume, plasticity and functionality are unknown. In our opinion, these data are paramount for better understanding the dose-response relationship between exercise and brain structure/function. We have shown that central fatigue was a major issue in long-distance running exercise (see, for example, [11]) yet, to the best of our knowledge, no studies have really assess cerebral alteration related to this type of exercise. This is because the observed decrease in voluntary activation does not mean that cortical alterations really occur, since peripheral changes, that is, the combination of influences including excitatory and inhibitory reflex inputs from muscles, joints, tendons and cutaneous afferents, may inhibit central drive at the spinal and supraspinal levels.

Also of interest is the investigation into pain perception and the possibility to describe interindividual differences in mechanisms of coping. Hormonal mechanisms (for example, cortisol) and neurotransmitters (for example, tryptophan, serotonin) are known to modulate the pain perception [12]. But most previous studies were limited to a single pain stimulus, whereas in the study of Schütz *et al. *[2] the stimuli are different among subjects and also fluctuating. The possibility of crossvalidation between the MRI, the psychometric and the biological results is promising for better describing the time course of factors influencing the fluctuation of pain throughout the race.

## Future directions and conclusions

This study provides the opportunity to explore the adaptive responses of humans submitted to the extreme load and stress induced by a 4,487-km road race. The methods used will allow investigation into various subsystems and their interaction in terms of tissue degeneration/regeneration, pain coping or cerebral adaptations. Future research directions can combine additional techniques such as transcranial magnetic stimulation (to assess cortical excitability and inhibition and supraspinal voluntary activation), cerebral multichannel near-infrared spectroscopy (to measure tissue hemodynamics), and electroencephalography or cerebral MRI, the latter in particular to assess long term cerebral alterations as in Schütz *et al. *[2].

It is uncertain if/how the findings in Schütz *et al. *paper can be translated to the fields of pathophysiology or critical illness, since the stress induced by the running load is highly specific. However, one may assume that some of the scientific knowledge accumulated will help in better understanding of adaptive responses.

Since it is a road stage race, it is likely that the adaptive responses to fatigue would be largely different in other environments/conditions such as high altitude, heat, mountainous competition or sleep deprivation. The exploration of exercising in such 'extreme environments' that often cannot be performed in a laboratory is an extending field of sports physiology or sports medicine. Together with large epidemiological surveys on ultramarathon runners that still have to be conducted, the amazing experiment of Schütz *et al. *[2] represents a unique attempt to investigate the limits of adaptive response of human bodies.

## Competing interests

The authors declare that they have no competing interests.

## Authors' contributions

GPM and GYM drafted the manuscript and gave final approval for publication.

## Pre-publication history

The pre-publication history for this paper can be accessed here:

http://www.biomedcentral.com/1741-7015/10/77/prepub
